# Impact of Plasma Electron Flux on Plasma Damage‐Free Sputtering of Ultrathin Tin‐Doped Indium Oxide Contact Layer on *p*‐GaN for InGaN/GaN Light‐Emitting Diodes

**DOI:** 10.1002/advs.201700637

**Published:** 2017-12-19

**Authors:** Kwang Jeong Son, Tae Kyoung Kim, Yu‐Jung Cha, Seung Kyu Oh, Shin‐Jae You, Jae‐Hyun Ryou, Joon Seop Kwak

**Affiliations:** ^1^ Department of Printed Electronics Engineering Sunchon National University Jeonnam 57922 Korea; ^2^ Department of Mechanical Engineering and Texas Center for Superconductivity at UH (TcSUH) University of Houston Houston TX 77204‐4006 USA; ^3^ Department of Physics Chungnam National University Daejeon 34134 Korea; ^4^ Materials Science and Engineering Program University of Houston Houston TX 77204 USA

**Keywords:** damage‐free sputter, light‐emitting diode, ohmic contacts, plasma electron flux, transparent conductive electrodes

## Abstract

The origin of plasma‐induced damage on a ***p***‐type wide‐bandgap layer during the sputtering of tin‐doped indium oxide (ITO) contact layers by using radiofrequency‐superimposed direct current (DC) sputtering and its effects on the forward voltage and light output power (LOP) of light‐emitting diodes (LEDs) with sputtered ITO transparent conductive electrodes (TCE) is systematically studied. Changing the DC power voltage from negative to positive bias reduces the forward voltages and enhances the LOP of the LEDs. The positive DC power drastically decreases the electron flux in the plasma obtained by plasma diagnostics using a cutoff probe and a Langmuir probe, suggesting that the repulsion of plasma electrons from the ***p***‐GaN surface can reduce plasma‐induced damage to the ***p***‐GaN. Furthermore, electron‐beam irradiation on ***p***‐GaN prior to ITO deposition significantly increases the forward voltages, showing that the plasma electrons play an important role in plasma‐induced damage to the ***p***‐GaN. The plasma electrons can increase the effective barrier height at the ITO/deep‐level defect (DLD) band of ***p***‐GaN by compensating DLDs, resulting in the deterioration of the forward voltage and LOP. Finally, the plasma damage‐free sputtered‐ITO TCE enhances the LOP of the LEDs by 20% with a low forward voltage of 2.9 V at 20 mA compared to LEDs with conventional e‐beam‐evaporated ITO TCE.

## 1 Introduction

Visible light‐emitting diodes (LEDs) based on indium gallium nitride/gallium nitride (InGaN/GaN) heterostructures are widely adopted in many applications, such as attractive solid‐state lighting sources for general lighting, vehicle illumination, displays, horticultural lighting, and hand‐held devices, thanks to their advantageous characteristics of low energy consumption, design flexibility, and long lifespans.^1^, ^2^, ^3^, ^4^, ^5^ Although blue LEDs yield excellent internal quantum efficiencies higher than 80% resulting from the continuous improvements in epitaxial materials,^6^ other LEDs have quantum efficiencies that still have margins for further improvement. Some critical areas are light absorption, electrical property, and interface quality of transparent conductive electrode (TCE). Tin‐doped indium oxide (In_2_O_3_:Sn or indium–tin oxide, ITO) deposited by electron‐beam (e‐beam) evaporation is the most commonly used TCE on a *p*‐GaN layer in InGaN/GaN LEDs. However, ITO still hampers further improvement of the light extraction efficiency. An interfacial contact layer formed between *p*‐GaN and ITO partially absorbs the light before it escapes from the epitaxial structure. Furthermore, a relatively thick ITO layer (≈200 nm) that is often formed to compensate for the high sheet resistance of the material degrades the optical transmittance of the TCE.

Many approaches have been reported to mitigate the light absorption in the interfacial contact layer by employing oxide interfacial layers,^7^, ^8^, ^9^ ultrathin metal interfacial layers,^10^, ^11^, ^12^, ^13^ and short‐period superlattice layers.^14^, ^15^ These techniques have demonstrated enhancements in the light output power (LOP). Additionally, hierarchical 33D nanostructured ITO contacts by oblique‐angle deposition (OAD) in e‐beam evaporation have been suggested to overcome light absorption by the e‐beam‐evaporated ITO layer.^16^, ^17^ Although the LEDs with 3D nanostructured ITO contacts exhibited a 29.5% enhancement in the LOP, these OAD techniques have a fundamental limitation for the fabrication of TCE because the ITO itself is so thick (typically thicker than 500 nm) that light absorption in the layers is still significant.

To overcome the limitations of ITO deposited by e‐beam evaporation, direct deposition by sputtering can be considered. The ITO layers deposited by sputtering show low sheet resistivity, a high optical transmittance at visible wavelengths, and a smooth surface; therefore, sputtering in place of e‐beam evaporation to fabricate ITO contacts is typical in many optical applications, such as touch‐sensitive screens, solar cells, and organic LEDs.^18^, ^19^, ^20^ In fact, sputtered ITO contacts on *p*‐GaN were found to be effective at reducing the light absorption because they did not contain the interfacial contact layer, and they can be deposited with a layer thinner than 100 nm. However, direct sputtering of ITO on *p*‐GaN has not been used in the fabrication of visible LEDs despite the advantageous optical properties because sputtering significantly increases the forward voltage of the LEDs due to plasma‐induced damage to the *p*‐GaN during the sputtering of ITO.^21^, ^22^, ^23^ Margalith et al. reported InGaN/GaN LEDs with direct‐current (DC)‐sputtered ITO contacts required an additional 2 V to drive 10 mA compared to similar devices with metal contacts.^21^ Chang et al. also showed that radiofrequency (RF)‐sputtered ITO contacts with in situ annealing increased the forward voltage at 20 mA by more than 1 V compared to that of the LEDs with metal contacts at the same current.^22^, ^23^ Recently, RF‐superimposed DC (RF+DC) sputtering was suggested for the direct sputtering of the ITO layer on *p*‐GaN.^24^, ^25^ The ITO contacts on *p*‐GaN by the new technique exhibited a low contact resistivity of ≈10^−2^ Ω cm^2^, which is low enough for a TCE on *p*‐GaN in visible LEDs. However, the studies did not investigate the mechanisms of plasma‐induced damage on *p*‐GaN during the sputtering, which is very important to minimize the forward operating voltage and maximize the LOP of the LEDs.

In this study, we systematically investigated the origin of plasma‐induced damage on *p*‐GaN in the LED structures during the sputtering of ITO to elucidate the mechanism of the forward voltage shift. Specifically, we studied the effect of DC power in the RF+DC sputtering of ITO on the forward voltage and LOP of InGaN/GaN LEDs because the DC power in the RF+DC sputtering can control the total flux of ions and electrons near the *p*‐GaN surface by controlling the discharge voltage and plasma potential using the ratio of the RF and DC power.^26^ The results show that the forward voltages of the LEDs with sputtered ITO contacts decrease drastically because the DC voltage changes from negative to positive bias. This behavior is a result of the repulsion of electrons from the *p*‐GaN surface by positive DC power, which is evidenced by plasma diagnostics using a cutoff probe and Langmuir probe. These findings were confirmed by the examination of electron beam irradiation on the *p*‐GaN in the LED structures prior to the deposition of ITO TCE.

## 2 Results

**Figure 1:**
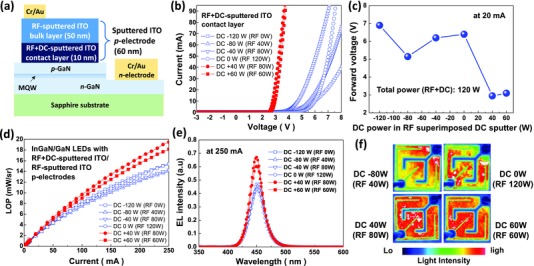
a) A schematic structure of sputtered ITO TCE consisting of an RF‐superimposed DC (RF+DC)‐sputtered ITO contact layer and an RF‐sputtered ITO layer. For the LEDs with the ITO contact layer deposited by RF+DC sputtering at various DC powers and annealed at 600 °C for 1 min, b) *I*–*V* curves, c) variation of forward voltages at 20 mA as a function of the DC power in RF+DC sputtering, d) LOP, e) EL intensity, and f) light intensity distribution images are displayed.

The InGaN/GaN blue LEDs were fabricated on c‐plane sapphire substrates with two‐step sputtered ITO TCE, which consisted of a thin ITO contact layer (10 nm) deposited on p‐GaN in the LED structures by RF+DC sputtering and a second ITO layer 50 nm thick deposited by RF sputtering, as schematically shown in **Figure** **1**a. The plasma discharge condition of the first contact layer was varied by changing the DC power in the RF+DC sputtering from −120 to +60 W, while the total power was kept at 120 W. The negative value of the DC power means that a negative bias was applied to the electrode. Figure 1b,c show the current–voltage (*I*–*V*) characteristics and the forward voltages at 20 mA, respectively, depending on the plasma discharge conditions. As the DC power was changed from negative to positive, the *I*–*V* characteristics drastically improved allowing higher currents at lower voltages. The forward voltage of the LEDs with ITO deposited by the DC power of −120 W (RF power of 0 W) was as high as 6.9 V at 20 mA. A change in the DC power in the negative range (−80 and −40 W) still resulted in high forward voltages over 5 V. The LEDs with ITO deposited with only an RF power of 120 W (DC power of 0 W) also exhibited a high forward voltage of 6.4 V. However, when the DC power was positive for the deposition of the ITO, the forward voltages of the LEDs significantly decreased. The forward voltages of the LEDs with ITO deposited using DC powers of +40 and +60 W (RF powers of 80 and 60 W, respectively) were 2.95 and 3.1 V at 20 mA, respectively. The forward voltages of the LEDs were critically dependent on the DC power in the RF+DC sputtering of the ITO contact layer, and they drastically decreased from over 5 to ≈3 V at 20 mA with a change in the DC power from negative to positive. As shown in Figure 1d,e, the LOP and electroluminescence (EL) intensity were also greatly improved for the LEDs with ITO deposited using positive DC powers. More than a 20% enhancement was observed for the EL peak intensity at 250 mA. This is attributed to a uniform distribution of light intensity, as shown in Figure 1f, owing to the low forward voltage and uniform current distribution in the ITO and p‐GaN layers. The light intensity was not uniform for the LEDs with the ITO deposited using negative or zero DC powers. These results suggest that the plasma‐induced damage is sensitive to the DC power in RF+DC sputtering of ITO and a positive DC power can prevent damage to the p‐GaN.

**Figure 2:**
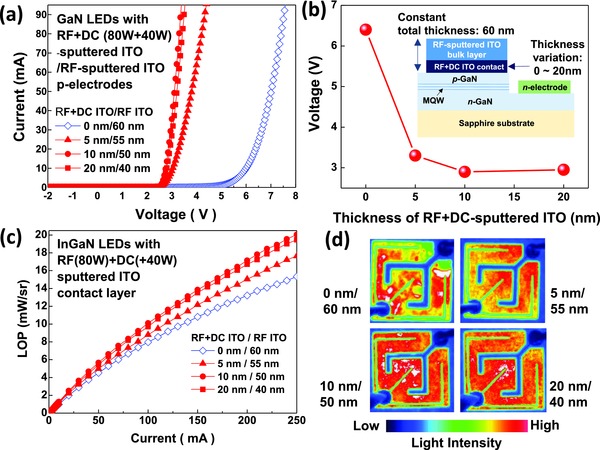
For the LEDs with various thicknesses of the RF+DC‐sputtered ITO contact layer annealed at 600 °C for 1 min, a) *I*–*V* characteristics, b) variation of forward voltages as a function of thickness of the RF+DC‐sputtered ITO contact layer (inset shows a schematic structure of LEDs with the various thickness of RF+DC‐sputtered ITO contact layer), c) LOP, and d) light intensity distribution images are displayed.

To further investigate the plasma‐induced damage, the thickness of the first ITO contact layer on p‐GaN in the LED structures was varied from 0 to 20 nm, while the total thickness of the two‐step ITO was maintained at 60 nm. The DC power in the RF+DC sputtering of the ITO contact layer was also kept at +40 W. As shown in **Figure** **2**a,b, as the thickness of the first ITO contact layer on p‐GaN increased from 0 to 5 nm, the forward voltages at 20 mA significantly decreased from 6.4 to 3.3 V. Additionally, the LOP of the LEDs at 250 mA was enhanced by 15%, as shown in Figure 2c. Further increases in the thickness to 10 and 20 nm reduced the forward voltage to 2.9 and 2.95 V, respectively. The LOPs of the LEDs were also further enhanced by 14% and 10% for 10 and 20 nm, respectively, compared to that of the LEDs with the 5 nm thick ITO contact layer, which is also attributed to the more uniform distribution of the light intensity (Figure 2d). These results imply that a certain thickness of the first ITO contact layer is required to protect the p‐GaN in the LED structures from plasma‐induced damage during RF sputtering of the second ITO layer. Although the 5 nm thick RF+DC‐sputtered ITO contact layer mitigated the damage on the surface of p‐GaN, it was not enough to eliminate it. However, RF+DC‐sputtered ITO contact layers thicker than 10 nm can protect the p‐GaN surface from the plasma‐induced damage.

**Figure 3:**
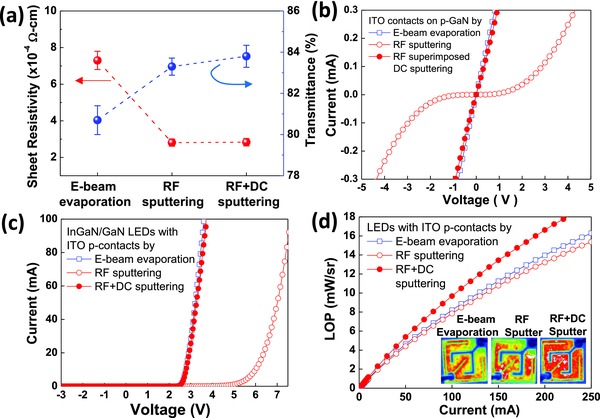
a) Variation of the sheet resistivity and transmittance of the ITO films after annealing at 600 °C for 1 min deposited by various methods (the data were obtained from 10 samples for each deposition method), b) *I*–*V* curves of the ITO contacts to *p*‐GaN deposited by various methods and annealed at 600 °C for 1 min, c) *I*–*V* characteristics of the LEDs with ITO deposited by various methods and annealed at 600 °C for 1 min, and d) LOPs of the LEDs with ITO deposited by various methods and annealed at 600 °C for 1 min (inset shows light intensity distribution images for the LEDs) are shown.

**Figure** **3** shows a comparison of TCE with the same thicknesses fabricated with conventional e‐beam‐evaporated ITO, RF‐sputtered ITO, and two‐step‐sputtered ITO, which was developed in this study, with the first contact layer (10 nm) using RF+DC (80 + 40 W) followed by the second RF‐sputtered layer (50 nm). As shown in Figure 3a, the RF‐sputtered ITO and the RF+DC‐sputtered ITO exhibited higher transmittance over 83% at 450 nm and a significantly lower sheet resistivity of 2.8 × 10^−4^ Ω cm than those of the e‐beam‐evaporated ITO, showing superior optical and electrical properties of the sputtered ITO films. However, the RF‐sputtered ITO on p‐GaN yielded nonlinear *I*–*V* characteristics of the LED structures, whereas the e‐beam‐evaporated ITO and two‐step‐sputtered ITO showed an ohmic contact behavior with linear *I*–*V* characteristics, as shown in Figure 3b. As a result, the forward voltage of the LEDs with RF‐sputtered ITO was as high as 6.4 V at 20 mA, but they were lower than 3 V for the LEDs with the e‐beam‐evaporated and two‐step‐sputtered ITO films, as shown in Figure 3c. The two‐step‐sputtered ITO contact exhibited similar electrical contact properties to the conventional e‐beam‐evaporated ITO contact, suggesting that plasma‐induced damage on the p‐GaN surface was prevented by creating an RF+DC‐sputtered ITO contact layer with positive DC power. The LOP of the LEDs with the two‐step sputtered ITO was enhanced by 20% at 250 mA than that of the LEDs with the e‐beam‐evaporated ITO, as shown in Figure 3d, which is attributed to the higher optical transmittance, lower sheet resistivity, and more uniform distribution of the light intensity, as shown in the insets of Figure 3d. Notably, a plasma‐induced damage‐free ITO TCE by sputtering was successfully demonstrated.

## 3 Discussion

**Figure 4:**
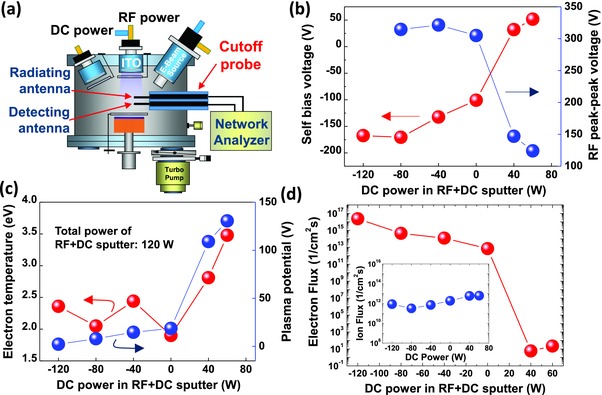
a) A schematic illustration of the RF+DC sputtering chamber and cutoff probe having two antenna, b) the variation of the self‐bias voltages and RF peak‐to‐peak voltages as a function of DC power in the RF+DC sputtering of ITO, c) the variation of the electron temperatures and plasma potentials as a function of the DC power in the RF+DC sputtering of ITO, and d) variation of electron fluxes as a function of the DC power in the RF+DC sputtering of ITO; the inset shows the variation of the ion fluxes as a function of DC power in the RF+DC sputtering of ITO.

To better understand the observed results and to study the origin of plasma‐induced damage during the sputtering of the ITO contacts on p‐GaN in the LED structures, we conducted plasma diagnostics using a cutoff probe and a single Langmuir probe.^27^, ^28^ As schematically shown in **Figure** **4**a, the cutoff probe, consisting of two coaxial cables with their cores exposed to and immersed in the plasma (the radiating antenna and the detecting antenna), measures the transmission frequency spectrum between the two antennas using a network analyzer. As shown in Figure 4b, the self‐bias voltage of the plasma generated by RF+DC sputtering increased gradually from −168 to −101 V, as the DC power was changed from −120 to 0 W. However, when the DC power was changed from 0 W to a positive power of +40 W, the self‐bias voltage increased rapidly to 33 V. Additionally, the RF peak‐to‐peak voltage dropped rapidly from 305 to 147 V as the DC power was increased from 0 to +40 W, whereas it did not change significantly when the DC power was increased in the negative range from −80 to 0 W. Furthermore, as shown in Figure 4c, the plasma potential increased rapidly from 15 to 109 V and 131 V as the DC power was increased from 0 W to the positive powers of +40 and +60 W, respectively, whereas it increased gradually from 2 to 15 V when the DC power was increased in the negative range from −120 to 0 W. Figure 4c also shows that the electron temperature increased rapidly from 1.9 to 3.5 V when the DC power was increased from 0 to +60 W. These results clearly show that positive DC power in the RF+DC sputtering of ITO causes significant changes in the plasma parameters. The plasma discharge condition can be altered significantly using a positive bias.

From the measurements, the electron flux (Γ_*e*_) was calculated using the following equation^29^

(1) $$\Gamma_{e}\;=\;\frac{1}{4}\bar{v}n_{0}e^{-\frac{V_{p}}{T_{e}}}$$

where *V*_p_ is the plasma potential, *T_e_* is the electron temperature, and $\bar{v}$ is mean electron speed that can be calculated from the following equation

(2) $$\bar{v}\;=\;{\left(\frac{8eT_{e}}{\pi m}\right)}^{\frac{1}{2}}\;\approx \;6.7\times 10^{7}\sqrt{T_{e}}$$

The ion flux (Γ_i_) was also calculated from the measurement using the following equation^29^

(3) $$\Gamma_{i}\;=\;\frac{1}{4}u_{B}n_{\mathrm{es}}\;=\;\frac{1}{4}{\left(\frac{eT_{e}}{M}\right)}^{\frac{1}{2}}0.61n_{e}$$

where *u*_B_ and *n*_es_ are the Bohm velocity and electron density on the sheath edge, respectively, and *M* is the ion mass of the Ar. The calculated electron and ion fluxes as a function of the DC power in the RF+DC sputtering of ITO are depicted in Figure 4d. The electron flux decreased gradually from 2.3 × 10^16^ to 7.0 × 10^12^ cm^−2^ s^−1^, when the DC power was changed in the negative range. As the DC power became positive, however, the electron flux was drastically reduced to the order of 10^1^ cm^−2^ s^−1^. The ion flux increased slightly within one order of magnitude, as the DC power was changed from positive to negative values, as shown in the inset of Figure 4d. This is the result of the rapid increase in the plasma potential near the ITO target as the DC power changes from negative to positive power, which drives the plasma electrons from the p‐GaN to the ITO target, resulting in the near‐complete removal of electrons near the p‐GaN surface (see S1 in the Supporting Information). It is worth noting that the positive DC power in the RF+DC sputtering of ITO can significantly repulse electrons in the plasma from the p‐GaN surface of the InGaN/GaN LEDs. The comparison between the variation of the forward voltages (Figure 1b) and electron flux (Figure 4d) as a function of the DC power in the RF+DC sputtering of ITO suggests that the plasma electrons play an important role in damaging the p‐GaN surface.

**Figure 5:**
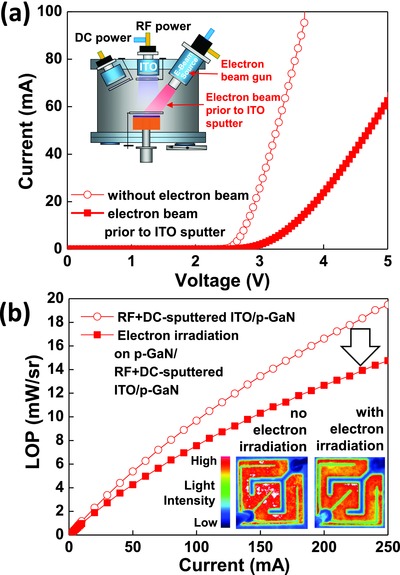
a) *I*–*V* characteristics of the LEDs with and without the electron beam irradiation on *p*‐GaN prior to the RF+DC‐sputtered ITO contact layer (inset shows a schematic illustration of the RF+DC sputter chamber equipped with a 2 in. electron‐beam gun), b) LOP of the LEDs with and without the electron beam irradiation on *p*‐GaN prior to sputtering the RF+DC‐sputtered ITO contact layer (inset shows the light intensity distribution image for the LEDs with and without electron beam irradiation).

To further investigate the origin of the plasma‐induced damage on *p*‐GaN in the LED structures, electron‐beam irradiation on the *p*‐GaN surface was applied prior to the deposition of the two‐step‐sputtered ITO contact layer. The DC power in the RF+DC sputtering was +40 W. Electron‐beam irradiation on *p*‐GaN can independently examine the role of the electrons on plasma‐induced damage on the *p*‐GaN surface. A 2 in. electron‐beam gun in the sputtering system, as shown in the inset of **Figure** **5**a, was used in which the electron‐beam was generated with an RF power of 150 W and a DC power of 100 V. The same two‐step ITO without electron‐beam irradiation was compared. It is worthy to note that the electron energy of the electron‐beam was comparable to that of the plasma electron of the RF sputtering of ITO (see S2 in the Supporting Information). The forward voltage of the LEDs with two‐step‐sputtered ITO was 2.9 V at 20 mA; however, it significantly increased to 4 V for the LEDs with the same two‐step ITO deposited after electron‐beam irradiation on *p*‐GaN, as shown in Figure 5a. Furthermore, Figure 5b shows that electron‐beam irradiation on *p*‐GaN prior to the two‐step‐sputtered ITO contact layer deposition drastically decreased the LOP of the LEDs, due to the nonuniform distribution of the light intensity, as shown in the insets of Figure 5b. The same results were also observed when the electron beam irradiation on *p*‐GaN occurred prior to the deposition of e‐beam‐evaporated ITO (see S2 in the Supporting Information).

These results clearly show that the electrons in the plasma can cause damage to the *p*‐GaN in the LED structures during the sputtering of ITO. The plasma electrons during RF+DC sputtering of ITO with negative or zero DC power causes plasma‐induced damage to the *p*‐GaN, resulting in a drastic increase in the forward voltage and a significant decrease in the LOP (Figure 1). Furthermore, RF+DC sputtering of ITO with a positive DC power can prevent plasma‐induced damage to the *p*‐GaN by the repulsion of electrons in the plasma due to the high plasma potential at the *p*‐GaN surface, as shown in Figure 4d, resulting in a low forward voltage of 2.9 V at 20 mA and an enhanced LOP by more than 30% compared to LEDs with the RF sputtered ITO (Figures 1 and 2).

Although the precise physical mechanism for the generation of plasma‐induced damage to the *p*‐GaN by the plasma electrons during the sputtering of ITO is not clearly understood at this moment, a possible explanation is the generation of the plasma‐induced damage in the deep level defect (DLD) band in *p*‐GaN by the plasma electrons during the sputtering of ITO. Many researchers have reported that the carrier transport at the metal/*p*‐GaN interface could be dominated by the DLD band in *p*‐GaN, instead of in the valence band of the *p*‐GaN. Kwak et al. suggested that defect‐assisted tunneling of carriers at the Pd/*p*‐GaN interface through the DLD bands may be present rather than Schottky barriers.^30^, ^31^ Park and Kim^32^ and Cha et al.^33^ also reported that carrier transport at the Ag/*p*‐GaN interface was dominated by the DLD bands in *p*‐GaN, resulting in low contact resistivities of 7 × 10^−4^ and 2 × 10^−3^ Ω cm^2^ in their studies, respectively. DeLucca et al. reported that the electrical properties of the metal contacts were strongly influenced by the presence of electrically active defects introduced during metal deposition on p‐GaN as well as n‐GaN.^34^, ^35^ Recently, Cha et al. reported that the carrier transport at the ITO/*p*‐GaN interface occurred through the DLD band in *p*‐GaN, where the effective barrier height between the ITO and the DLD band in *p*‐GaN was as low as 0.12 eV.^24^ Furthermore, Oh et al. reported that the electron‐beam irradiation on the source‐to‐gate and gate‐to‐drain of the AlGaN/GaN heterostructure field‐effect transistors reduced leakage currents due to a decrease in the surface trap density, suggesting the neutralization of the defect traps by the electron‐beam irradiation.^36^

**Figure 6:**
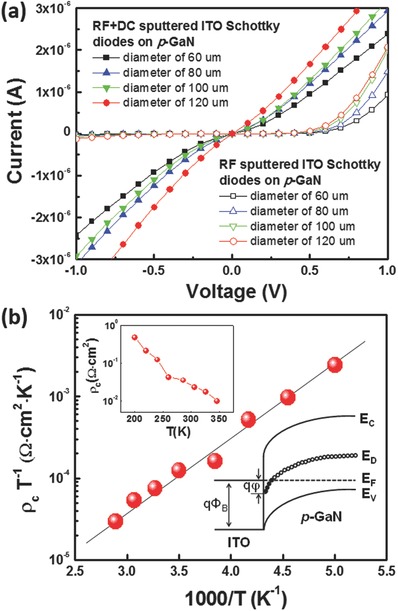
a) *I*–*V* characteristics of the RF+DC‐sputtered ITO Schottky diodes and RF‐sputtered ITO Schottky diodes on *p*‐GaN, where the Ni/Ag contacts were used as ohmic contacts on *p*‐GaN, and b) the variation of ρ_c_T ^− 1^ occurred as a function of 1000/*T*. The insets show the ρ_c_–T plots and the schematic band diagram if the ITO contact on *p*‐GaN at zero bias.

To understand the role of the plasma electrons on the generation of the plasma‐induced damage in the DLD band in *p*‐GaN, we fabricated Schottky diodes on *p*‐GaN with RF‐sputtered ITO, and two‐step‐sputtered ITO with a first contact layer (10 nm) using RF+DC sputter followed by a second RF‐sputtered layer (50 nm). Four sputtered ITO Schottky diodes with different diameters were fabricated on *p*‐GaN in the LED structures in which Ni/Ag (2 nm/200 nm) was used as an ohmic contact on *p*‐GaN (see S4 in the Supporting Information) As shown in **Figure** **6**a, RF+DC‐sputtered ITO Schottky diodes on *p*‐GaN in the LED structure resulted in nearly linear *I*–*V* behavior, and the *I*–*V* curves became more linear as the diameter of the RF+DC‐sputtered ITO Schottky diodes on *p*‐GaN increased. On the other hand, the RF‐ sputtered ITO Schottky diodes on *p*‐GaN showed rectifying characteristics, as shown in Figure 6a. These behaviors are quite similar to the previous report. Yu et al. investigated the origin of the leaky characteristics of Schottky diodes on p‐GaN and reported that the Ni Schottky contacts on *p*‐GaN exhibited quasi‐ohmic behavior due to the carrier transport with significant tunneling across a highly defective *p*‐GaN surface layer. Additionally, the *I*–*V* characteristics of the Ni Schottky contacts on *p*‐GaN improved when the thin defective *p*‐GaN surface layer was etched by reactive ion etching,^37^ indicating that the defective *p*‐GaN surface layer plays an important role in the leaky characteristics of Schottky diodes on *p*‐GaN. Furthermore, the Schottky barrier heights for the RF‐sputtered ITO Schottky diodes on *p*‐GaN were obtained as 0.72 eV for the four diameters (60, 80, 100, 120 µm) of the RF‐sputtered ITO Schottky diodes, indicating the formation of good Schottky diodes by RF‐sputtered ITO on *p*‐GaN. This agrees with a previous report by Lin et al. in which excellent ITO Schottky diodes on *p*‐GaN were fabricated using the RF‐sputtered ITO.^38^

However, we could not calculate the barrier height of the RF+DC‐sputtered ITO contacts on *p*‐GaN from the Schottky diodes because of the significant leaky Schottky diode characteristics, as shown in Figure 6a. To obtain the barrier height at the RF+DC‐sputtered ITO/*p*‐GaN interface, we performed temperature‐dependent contact resistivity measurements for the RF+DC‐sputtered ITO contacts of *p*‐GaN in the LED structures. Considering the dominant current flow at the ITO/*p*‐GaN interface through a DLD band rather than the valence band,^30^, ^32^, ^33^ we can estimate the effective barrier height (*qϕ*) at the RF+DC‐sputtered ITO/*p*‐GaN interface, as defined in the inset in Figure 6b, using the relationship between the contact resistivity and defect density,^39^ which is given by the following equation

(4) $$\rho_{C}\;=\;\frac{k_{B}T}{q^{2}k\;\Theta_{D}/h\;\exp\left(-\gamma /aN_{\mathrm{DE}}^{1/3}\right){\left(3/4\pi N_{\mathrm{DE}}\right)}^{1/3}N_{\mathrm{DE}}}\exp\left(\frac{q\varphi }{kT}\right)$$

where Θ_D_ is the Debye temperature, γ is a constant, *a* is the extent of the wave function, and *N*_DE_ is the defect density. From the linear relationship between ρ_c_T^−1^ and 1000/*T*, the *qϕ* was calculated to be 0.18 eV, which is low enough to produce the low contact resistivity of the RF+DC‐sputtered ITO ohmic contacts to *p*‐GaN. It is worthy to note that the obtained effective barrier height of the RF+DC‐sputtered ITO contact to *p*‐GaN is similar to that of the e‐beam‐evaporated ITO contact to *p*‐GaN,^40^ suggesting that the elimination of plasma electrons from the *p*‐GaN using the RF+DC sputtering of ITO can prevent the thin defective *p*‐GaN surface layer from the plasma damage during the sputtering of ITO, followed by the low effective barrier height at the sputtered ITO/*p*‐GaN interface.

The significantly higher barrier height of the RF‐sputtered ITO contacts on *p*‐GaN (0.72 eV) compared to that of the RF+DC‐sputtered ITO contacts on *p*‐GaN (0.18 eV) strongly suggests that the plasma electrons during the RF sputtering of ITO can counteract the DLDs in the *p*‐GaN surface and reduce the junction leakage due to the decreased carrier tunneling through the DLDs at the ITO/*p*‐GaN interface, because the barrier height at the metal/*p*‐GaN is closely related to the DLDs in *p*‐GaN. Shiojima et al. reported that the barrier height at the Ni/*p*‐GaN increased from 0.86 to 2.45 eV, as the deep level defects were filled by illumination by white light, which was attributed to that the tunneling current at the metal/*p*‐GaN was impeded.^41^ Furthermore, the defect density of *p*‐GaN surface at the RF+DC‐sputtered ITO/*p*‐GaN was calculated as 1.6 × 10^19^ cm^−3^ from Equation (5), while that of *p*‐GaN surface at the RF‐ sputtered ITO/*p*‐GaN was significantly reduced to 0.8 × 10^19^ cm^−3^. Therefore, the plasma electrons during the RF+DC sputtering of ITO with negative or zero DC power may counteract the DLDs in the *p*‐GaN surface and reduce the density of DLDs, which can increase the effective barrier height at the ITO/*p*‐GaN. Conversely, plasma‐induced damage‐free ITO sputtered using positive DC power can be realized for InGaN/GaN LEDs by the repulsion of electrons near the *p*‐GaN surface.

**Figure 7:**
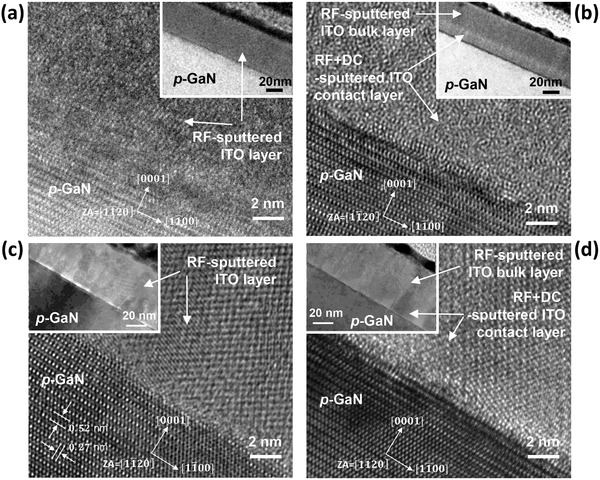
a) A high‐magnification cross‐sectional TEM image of as‐deposited RF‐sputtered ITO on *p*‐GaN (inset shows the low‐magnification TEM image), where the zone axis (ZA) is $\left[1\bar{1}20\right]$, b) a high‐magnification cross‐sectional TEM image of an as‐deposited RF+DC‐sputtered ITO contact layer on *p*‐GaN (inset shows the low‐magnification TEM image), c) a high‐magnification cross‐sectional TEM image of an annealed RF‐sputtered ITO on *p*‐GaN (inset shows the low‐magnification TEM image), and d) a high‐magnification cross‐sectional TEM image of an annealed RF+DC‐sputtered ITO contact layer on *p*‐GaN (inset shows the low‐magnification TEM image).

Finally, to investigate the effect of the plasma electrons on the microstructure of the sputtered ITO layer on *p*‐GaN, cross‐sectional transmission electron microscopy (TEM) was performed. The samples include RF‐sputtered ITO and two‐step‐sputtered ITO sputtered with positive DC power for the first contact layer. As shown in **Figure** **7**a, the as‐deposited RF‐sputtered ITO showed a crystalline microstructure with small grains at the ITO/*p*‐GaN interface and a single layer structure with a smooth surface. However, the two‐step‐sputtered ITO was different, suggesting different microstructures, as shown in the inset of Figure 7b. The high‐resolution TEM image of the first contact layer in the two‐step‐sputtered ITO exhibited an amorphous microstructure. After annealing at 600 °C for 1 min, however, the two‐step‐sputtered amorphous ITO contact layer transformed to exhibit crystalline microstructures, as shown in Figure 7d, which was identical to the microstructures of the RF‐sputtered ITO, as shown in Figure 7c. Additionally, both layers of two‐step‐sputtered ITO exhibited the same properties, indicating the same crystalline microstructures after annealing. The difference between the crystal structures of the as‐deposited RF‐sputtered ITO and the two‐step‐sputtered ITO contact layer could be related to the difference in the plasma discharge conditions, especially in the electron flux on *p*‐GaN. The electron bombardment during the film growth can increase the rearrangement of ad‐atoms of ITO on *p*‐GaN, followed by crystallization of the sputtered ITO film. Wie et al. reported that in situ irradiation of the electron beam during the deposition of thin ITO films at room temperature produced fully crystallized thin ITO films on glass substrates through the energy transfer from the irradiated electrons to ad‐atoms of the ITO.^42^ On the other hand, the as‐deposited RF+DC‐sputtered ITO contact layer exhibited an amorphous microstructure due to the absence of the plasma electrons with applied positive DC power. It is worth noting that the as‐deposited RF+DC‐sputtered amorphous ITO showed a similar crystal structure to that of the as‐deposited e‐beam‐evaporated ITO on *p*‐GaN (see S5 in the Supporting Information), which may imply that ad‐atoms of ITO on *p*‐GaN during the RF+DC sputtering of ITO with positive DC power may have a low energy.

Therefore, plasma‐induced damage during the sputtering of ITO may originate from the plasma electrons, which can yield the energetic ad‐atoms of ITO on *p*‐GaN during sputtering and may increase the effective barrier height at the ITO/DLD band of *p*‐GaN due to the compensation of DLDs in the *p*‐GaN surface. The RF+DC sputtering of ITO with positive DC power drastically reduced the electron flux on the *p*‐GaN surface during the sputtering of ITO, as shown in Figure 4d, and successfully demonstrated plasma‐induced damage‐free sputtering of ITO *p*‐electrodes, which resulted in a low forward voltage of 2.9 V at 20 mA and a 20% improved LOP of the InGaN/GaN LEDs compared to the LEDs with conventional e‐beam‐evaporated ITO *p*‐electrodes, as shown in Figure 3.

## 4 Conclusion

We systematically examined the origin of plasma‐induced damage to the *p*‐GaN surface during the sputtering of ITO TCE and its effects on the forward voltage and LOP of InGaN/GaN LEDs. First, we investigated the effect of DC power in RF+DC sputtering of ITO on the forward voltage and LOP of InGaN/GaN LEDs and found that the plasma‐induced damage was sensitive to the DC power. The forward voltages of the LEDs at 20 mA drastically decreased from over 5 to 3 V, and the LOP of the LEDs was greatly enhanced by more than 20% at 250 mA when the DC power was changed from negative to positive values. Second, the electron and ion fluxes during the RF+DC sputtering of ITO with the various DC power were calculated based on the plasma discharge parameters measured by a cutoff probe and a Langmuir probe. Changing the DC power to a positive bias drastically reduced the electron flux in the plasma, suggesting that the plasma electrons play an important role in the plasma‐induced damage of the *p*‐GaN surface. Furthermore, the significant increase in the forward voltage of the LEDs was observed, when electron‐beam irradiation was applied to the *p*‐GaN surface. This confirms that the plasma electrons, and not the ions, can cause plasma‐induced damage to the *p*‐GaN during the sputtering of ITO. Lastly, the physical mechanism for the generation of plasma‐induced damage on *p*‐GaN by the plasma electrons was suggested. The plasma electrons can counteract the DLDs in the *p*‐GaN surface and reduce the density of DLDs, which increases the effective barrier height at the ITO/DLD band of *p*‐GaN. Furthermore, the plasma electrons yielded energetic ad‐atoms of ITO on *p*‐GaN during sputtering through a transfer of energy from the electrons to the ad‐atoms and increased the plasma‐induced damage on *p*‐GaN. We successfully demonstrated a plasma‐induced damage‐free ITO TCE on InGaN/GaN LEDs by sputtering, which showed a 20% improvement in the LOP of the LEDs with a comparable forward voltage of 2.9 V at 20 mA compared to LEDs with conventional e‐beam‐evaporated ITO.

## 5 Experimental Section

*Fabrication of the InGaN/GaN LEDs with RF+DC‐Sputtered ITO Contact Layer*: The InGaN/GaN LED structures were grown on *c*‐plane sapphire substrates by metal‐organic chemical vapor deposition. Ammonia, trimethylgallium, trimethylaluminum, and trimethylindium were used as the precursors and bis(cyclopentadienyl)magnesium and silane were used as the dopants. The epitaxial InGaN/GaN LED structure consisted of a low‐temperature 30 nm thick GaN buffer layer, a 4 µm thick *n*‐type GaN layer, six pairs of InGaN/GaN multiple quantum wells, and a 200 nm thick *p*‐GaN layer. The *p*‐GaN layers consisted of three different Mg doping concentration regions as follows: 1 × 10^19^ cm^−3^ for the first 50 nm thick *p*‐GaN layer, 5 × 10^18^ cm^−3^ for the second 130 nm thick *p*‐GaN layer, and 1.5 × 10^20^ cm^−3^ for the 20 nm thick top *p*‐GaN layer. The first and second *p*‐GaN layers were designed to enhance the carrier concentration, and the top *p*‐GaN layer was designed to increase the density of defects in the DLD band of *p*‐GaN.^30^, ^31^ For the fabrication of lateral‐type LEDs, a mesa pattern with the dimensions of 500 × 500 µm^2^ was transferred into the *n*‐GaN layer down to 1.0 µm by inductively coupled plasma etching. After the mesa etching, a 60 nm thick ITO layer was deposited on *p*‐GaN using RF sputtering, e‐beam evaporation, and RF+DC sputtering, followed by annealing at 600 °C for 1 min in N_2_/O_2_ ambient air. Finally, Cr/Au (30/800 nm) were deposited on the *n*‐type GaN and ITO TCE as an *n*‐electrode and a contact pad, respectively.

*Measurement of Electron Flux by the Cutoff Probe and Single Langmuir Probe*: The cutoff probe was made of two coaxial cables in a stainless‐steel holder. The end two tips (the radiating antenna and the detecting antenna) of the cables were exposed to the plasma. A network analyzer was used to make a transmission spectrum and measure the transmission frequency spectrum between the two antennas. The transmission frequency spectra (S21), as defined by Equation (5), were obtained through a Fourier analysis of the transmitted impulse signals, and they were then used to measure the electron densities

(5) $$S21\;=\;10\;\times \;\log_{10}\left(\frac{P_{\mathrm{out}}}{P_{\mathrm{in}}}\right)$$

where *P*_in_ and *P*_out_ are the source signal power and a transmitted signal power, respectively. The Langmuir probe measurement was made to measure the electron temperature and plasma potential as well as to check the reliability of the cutoff probe.

*Measurement of the LOP of InGaN/GaN LEDs with RF+DC‐Sputtered ITO Contact Layer*: The light output power of InGaN/GaN LEDs with ITO TCE was measured at an on‐wafer level using an integrated sphere. The structural properties of the RF+DC‐sputtered ITO contact layer, as well as the RF‐sputtered ITO layer on *p*‐GaN, were characterized by using TEM (JEOL JEM‐2100F, 200 keV). Optical properties (transmittance and light distribution) were measured using a UV–vis spectrometer (Agilent, Cary 5000) and light beam profiler (Metrolux, ML‐3720).

## Conflict of Interest

The authors declare no conflict of interest.

## Supporting information

Supplementary

## References

[1] S. Pimputkar , J. S. Speck , S. P. DenBaars , S. Nakamura , Nat. Photonics 2009, 3, 180.

[2] I. Akasaki , Angew. Chem., Int. Ed. 2015, 54, 7750. 10.1002/anie.201502664 26012383

[3] X. Lu , C. Liu , H. Jiang , X. Zou , A. Zhang , K. M. Lau , Appl. Phys. Lett. 2016, 109, 053504.

[4] R. C. Morrow , HortScience 2008, 43, 1947.

[5] H. S. Lee , H. J. Park , J. S. Kwak , Appl. Opt. 2017, 56, 5106. 29047663 10.1364/AO.56.005106

[6] S. P. DenBaars , D. Feezell , K. Kelchner , S. Pimputkar , C.‐C. Pan , C.‐C. Yen , S. Tanaka , Y. Zhao , N. Pfaff , R. Farrell , M. Iza , S. Keller , U. Mishra , J. S. Speck , S. Nakamura , Acta Mater. 2013, 61, 945.

[7] J.‐O. Song , J. S. Kwak , Y. Park , T.‐Y. Seong , Appl. Phys. Lett. 2005, 86, 213505.

[8] S.‐M. Pan , R.‐C. Tu , Y.‐M. Fan , R.‐C. Yeh , J.‐T. Hsu , IEEE Photonics Technol. Lett. 2003, 15, 646.

[9] J.‐O. Song , J. S. Ha , T.‐Y. Seong , IEEE Trans. Electron Devices 2010, 57, 42.

[10] J.‐O. Song , D.‐S. Leem , J. S. Kwak , Y. Park , S. W. Chae , T.‐Y. Seong , IEEE Photonics Technol. Lett. 2005, 17, 291.

[11] J.‐O. Song , H.‐G. Hong , J.‐W. Jeon , J.‐I. Sohn , J.‐S. Jang , T.‐Y. Seong , Electrochem. Solid‐State Lett. 2008, 11, H36.

[12] L.‐C. Chen , S.‐F. Lu , Phys. Status Solidi A 2006, 203, 2451.

[13] S. W. Chae , K. C. Kim , D. H. Kim , T. G. Kim , S. K. Yoon , B. W. Oh , D. S. Kim , H. K. Kim , Y. M. Sung , Appl. Phys. Lett. 2007, 90, 181101.

[14] T. Gessmann , J. W. Graff , Y.‐L. Li , E. L. Waldron , E. F. Schubert , J. Appl. Phys. 2002, 92, 3740.

[15] S. J. Chang , C. S. Chang , Y. K. Su , R. W. Chuang , W. C. Lai , C. H. Kuo , Y. P. Hsu , Y. C. Lin , S. C. Shei , H. M. Lo , J. C. Ke , J. K. Sheu , IEEE Photonics Technol. Lett. 2004, 16, 1002.

[16] H. Kwon , S. H. Lee , J. K. Kim , Nanoscale Res. Lett. 2015, 10, 369. 26391174 10.1186/s11671-015-1057-2 PMC4577498

[17] M. J. Park , C. U. Kim , S. B. Kang , S. H. Won , J. S. Kwak , C.‐M. Kim , K. J. Choi , Adv. Opt. Mater. 2017, 5, 2.

[18] C.‐H. Hong , J.‐H. Shin , N.‐M. Park , K.‐H. Kim , B.‐S. Kim , J. S. Kwak , B.‐K. Ju , W.‐S. Cheong , Jpn. J. Appl. Phys. 2014, 53, 08NG01.

[19] K. A. Bush , C. D. Bailie , Y. Chen , A. R. Bowring , W. Wang , W. Ma , T. Leijtens , F. Moghadam , M. D. McGehee , Adv. Mater. 2015, 28, 3937. 10.1002/adma.201505279 26880196

[20] H.‐K. Kim , D.‐G. Kim , K.‐S. Lee , M.‐S. Huh , S. H. Jeong , K. I. Kim , T.‐Y. Seong , Appl. Phys. Lett. 2005, 86, 183503.

[21] T. Margalith , O. Buchinsky , D. A. Cohen , A. C. Abare , M. Hansen , S. P. DenBaars , L. A. Coldren , Appl. Phys. Lett. 1999, 74, 3930.

[22] C. S. Chang , S. J. Chang , Y. K. Su , Y. C. Lin , Y. P. Hsu , S. C. Shei , S. C. Chen , C. H. Liu , U. H. Liaw , Semicond. Sci. Technol. 2003, 18, L21.

[23] S. J. Chang , C. H. Lan , J. D. Hwang , Y. C. Cheng , W. J. Lin , J. C. Lin , H. Z. Chen , J. Electrochem. Soc. 2008, 155, H140.

[24] Y.‐J. Cha , G. J. Lee , Y. L. Lee , S. K. Oh , J. S. Kwak , Thin Solid Films 2015, 591, 182.

[25] T. K. Kim , Y. J. Yoon , S. K. Oh , Y. L. Lee , Y.‐J. Cha , J. S. Kwak , Appl. Surf. Sci. 2018, 432, 233.

[26] S. I. Kim , S. H. Cho , S. R. Choi , H. H. Yoon , P. K. Song , Curr. Appl. Phys. 2009, 9, S262.

[27] D. W. Kim , S. J. You , B. K. Na , J. H. Kim , H. Y. Chang , Appl. Phys. Lett. 2011, 99, 131502.

[28] S. J. You , S. S. Kim , H. Y. Chang , Appl. Phys. Lett. 2004, 85, 4872.

[29] M. A. Lieberman , A. J. Lichtenberg , Principles of Plasma Discharges and Materials Processing, Wiley, New York 1994, p. 188.

[30] J. S. Kwak , O.‐H. Nam , Y. Park , Appl. Phys. Lett. 2002, 80, 3554.

[31] J. S. Kwak , O.‐H. Nam , Y. Park , J. Appl. Phys. 2004, 95, 5917.

[32] Y. Park , H. Kim , Appl. Phys. Express 2011, 4, 085701.

[33] Y.‐J. Cha , S. K. Oh , J. S. Kwak , Mater. Lett. 2015, 158, 363.

[34] J. M. DeLucca , S. E. Mohney , F. D. Auret , S. A. Goodman , J. Appl. Phys. 2000, 88, 2593.

[35] J. M. DeLucca , H. S. Venugopalan , S. E. Mohney , R. F. Karlicek Jr ., Appl. Phys. Lett. 1998, 73, 3402.

[36] S. K. Oh , C. G. Song , T. Jang , J. S. Kwak , J. Semicond. Technol. Sci. 2013, 13, 617.

[37] L. S. Yu , L. Jia , D. Qiao , S. S. Lau , J. Li , J. Y. Lin , H. X. Jiang , IEEE Trans. Electron Devices 2003, 50, 292.

[38] Y. J. Lin , C.‐W. Hsu , J. Electron. Mater. 2004, 33, 1036.

[39] H. Yamamoto , Z. Fang , D. C. Look , Appl. Phys. Lett. 1990, 57, 1537.

[40] Y. Choi , H. Kim , J. Alloys Compd. 2012, 533, 15.

[41] K. Shiojima , T. Sugahara , S. Sakai , Appl. Phys. Lett. 2000, 77, 4353.

[42] S.‐M. Wie , C.‐H. Hong , S. K. Oh , W.‐S. Cheong , Y. J. Yoon , J. S. Kwak , Ceram. Int. 2014, 40, 11163.

