# Supplementary material for: Impact of Plasma Electron Flux on Plasma Damage‐Free Sputtering of Ultrathin Tin‐Doped Indium Oxide Contact Layer on p‐GaN for InGaN/GaN Light‐Emitting Diodes
Source: Adv Sci (Weinh). 2017 Dec 19;5(2):1700637. doi: 10.1002/advs.201700637 (PMC5827458; doi:10.1002/advs.201700637)
Supplement: Supplementary file 1 — Supplementary [file ADVS-5-1700637-s001.pdf]

Copyright WILEY-VCH Verlag GmbH & Co. KGaA, 69469 Weinheim, Germany, 2017.

ADVANCED SCIENCE
Open Access

Supporting Information

for *Adv. Sci.,* DOI: 10.1002/advs.201700637

Impact of Plasma Electron Flux on Plasma Damage-Free Sputtering of Ultrathin Tin-Doped Indium Oxide Contact Layer on *p*-GaN for InGaN/GaN Light-Emitting Diodes

*Kwang Jeong Son, Tae Kyoung Kim, Yu-Jung Cha, Seung Kyu Oh, Shin-Jae You, Jae-Hyun Ryou, and Joon Seop Kwak**

Supporting Information

Impact of plasma electron flux on plasma damage-free sputtering of ultrathin indium-tin-oxide contact layer on *p*-GaN for InGaN/GaN light-emitting diodes

By Kwang Jung Son, Tae Kyoung Kim, Yu-Jung Cha, Seung Kyu Oh, Shin-Jae You, Jae-Hyun Ryou and Joon Seop Kwak

S1. Variation of plasma discharge parameters as a function of DC power in the RF-superimposed DC sputtering of ITO

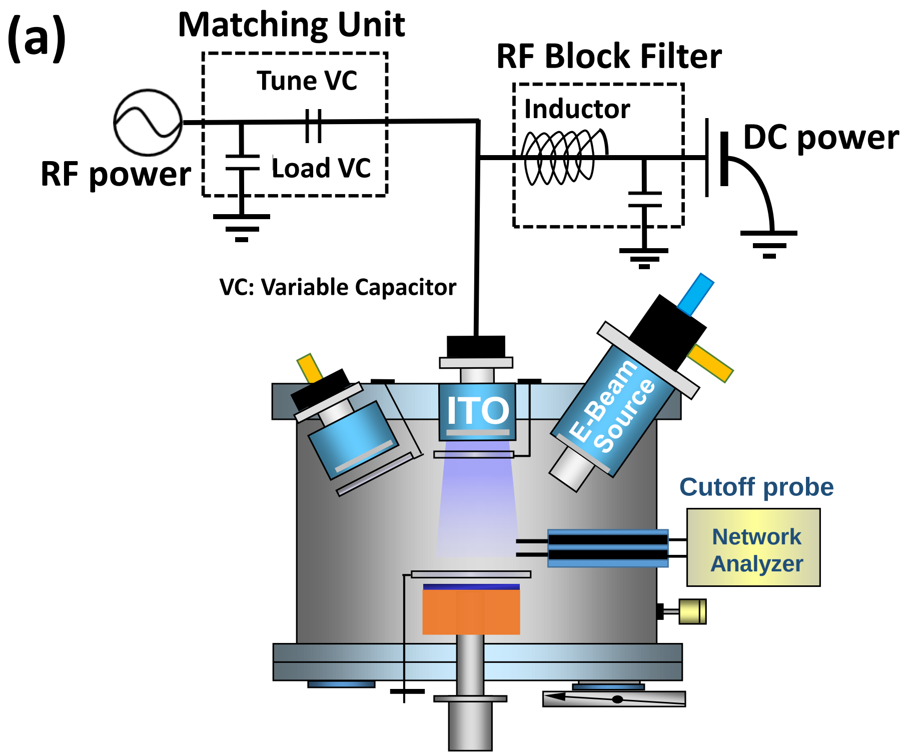

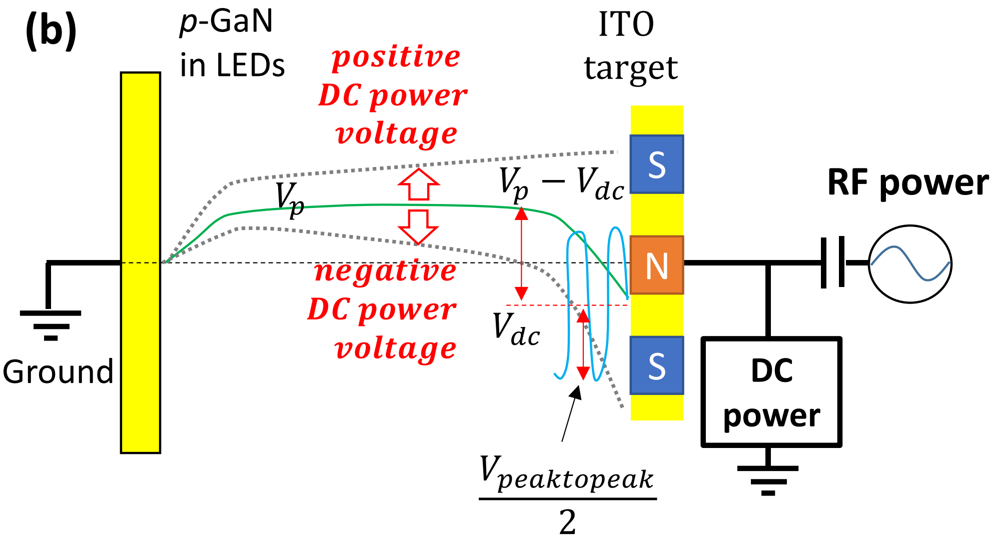

Figure S1. (a) A schematic illustration of the RF superimposed DC (RF+DC) sputtering system, and (b) a schematic illustrating the distribution of plasma potential

Table S1. Measured plasma discharge parameters from plasma diagnosis using cutoff probe and Langmuir probe.

Power (W)		Vp (V)	$\frac{V_{peaktopeak}}{2}$ (v)	Vdc (V)	$V_p - V_{dc}$ (V)	Debye length (mm)	Sheath width (mm)
DC power	RF power						
-120	0	2.3	-	-167.5	169.8	0.233	2.33
-40	80	14.8	160.8	-132.5	147.3	0.263	2.63
0	120	18.7	152.5	-100.8	119.5	0.135	1.35
40	80	109.1	73.5	32.1	77.0	0.102	1.02
60	60	130.6	62.0	51.7	78.9	0.117	1.17

In this study, as shown in Figure S1(a), RF-superimposed DC (RF+DC) sputtering was used for the direct sputtering of the ITO layer on *p*-GaN in the LED structures, which can significantly change the distribution of plasma potential according to the DC power, as schematically shown in Figure S1(b). In the RF superimposed DC sputter system used in this study, RF power was matched with the Tune variable

capacitor (VC) and Load VC, and it was superimposed on the DC power. Then, the RF superimposed DC power was applied to the electrode of ITO target. The inductor and capacitor in the RF Block Filter was designed to prevent the leakage of RF power.

For characterizing the plasma potential distribution, plasma discharge parameters were measured using a cutoff probe and a Langmuir probe, as summarized in Table S1. Furthermore, based upon the measurement of the plasma discharge parameters, a schematic illustration of the distribution of plasma potential was shown in Figure S1(b). As the DC power in the RF-superimposed DC sputtering of ITO is applied from negative to positive power, plasma potential near to ITO target increased rapidly, which accelerate plasma electrons having negative charge from the *p*-GaN surface to the ITO target, followed by nearly complete repulsion of plasma electrons from the *p*-GaN surface.

S2. Measurement of the electron density of the plasma electron during the RF sputtering of ITO and electron-beam irradiation using the cut-off probe

In order to compare the electron-beam energy to the plasma electron during the RF sputtering of ITO, we measured the electron density of the plasma electron during the RF sputtering of ITO as well as electron-beam irradiation using the cut-off probe. As shown in the following Figure S2, the density of plasma electron during the RF sputtering of ITO increased gradually from 1.3×10^9 cm^{-3} to 2.3×10^9 cm^{-3} as the RF power increased from 40 W to 160W. The density of plasma electron for the RF power of 120 W in this study was measured as 1.9×10^9 cm^{-3}. The electron density during the electron-beam irradiation as a function of DC power (RF power was kept as 150 W) in the electron-beam gun was also measured. As shown in the Figure S2, the electron density increased from 4.0×10^8 cm^{-3} to 1.6×10^9 cm^{-3} as the DC power in the electron-beam gun increased from 50 V to 1500 V. The electron density for the DC power of 1000 W in this study was measured as 1.4×10^9 cm^{-3}. These results show that the electron density of the electron-beam is similar to that of the RF sputtering of ITO in this study.

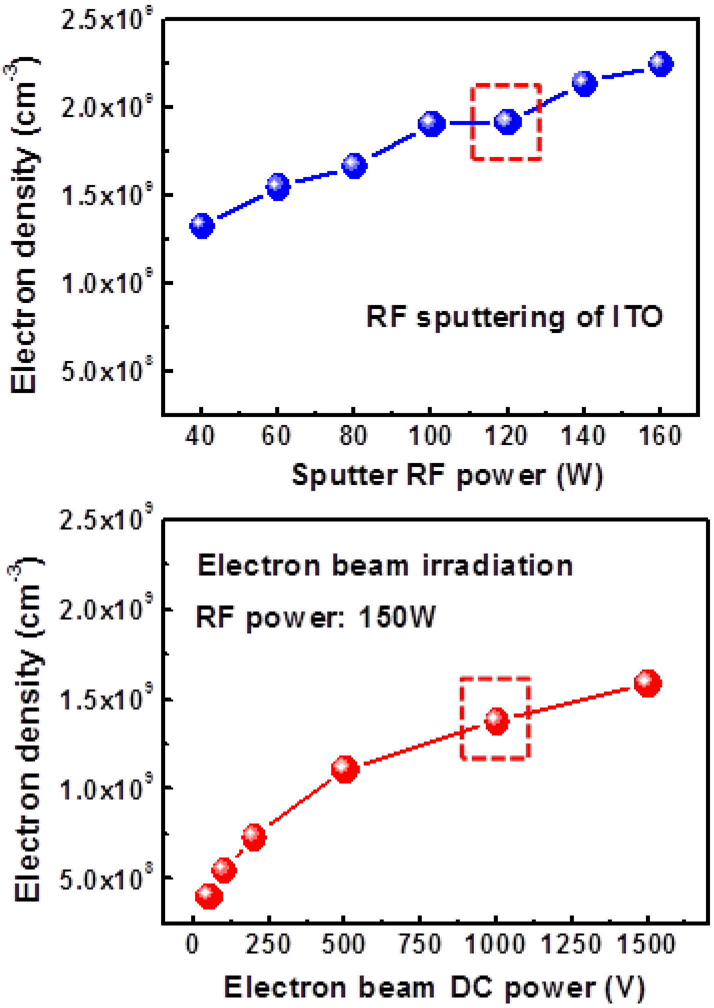

Figure S2. Variation of electron density as a function of (a) RF power in the RF sputtering of ITO and (b) DC power of electron-beam gun in the electron-beam irradiation on p-GaN.

S3. Electron-beam irradiation on *p*-GaN prior to e-beam evaporated ITO p-electrodes

We also examined the electron beam irradiation on *p*-GaN prior to the deposition of the e-beam evaporated ITO electrode. A 2-inch electron-beam gun in the sputtering system, as shown in the inset of Fig. 5(a), was used in which the electron-beam was generated with an RF power of 150 W and a DC power of 1 kV. For comparison, the e-

beam evaporated ITO electrode was deposited on *p*-GaN without the electron-beam irradiation from the 2-inch diameter electron-beam gun. As shown in Fig. S3(a), the forward voltage at 20 mA significantly increased to over 8 V for the LEDs with the electron beam irradiation on *p*-GaN prior to deposition of the e-beam-evaporated ITO electrode. As shown in Figs. S3(b) and (c), The LOP and EL intensity of the LEDs with the electron-beam irradiation on *p*-GaN prior to the deposition of the e-beam-evaporated ITO electrode were reduced, due to the non-uniform distribution of light intensity as shown in Fig. S3(d). These clearly demonstrate that electron-beam irradiation caused severe plasma-induced damage on *p*-GaN, and suggest that removal of electron flux during sputtering of ITO on *p*-GaN is essential to obtain plasma-induced damage-free sputtered ITO electrode on p-GaN.

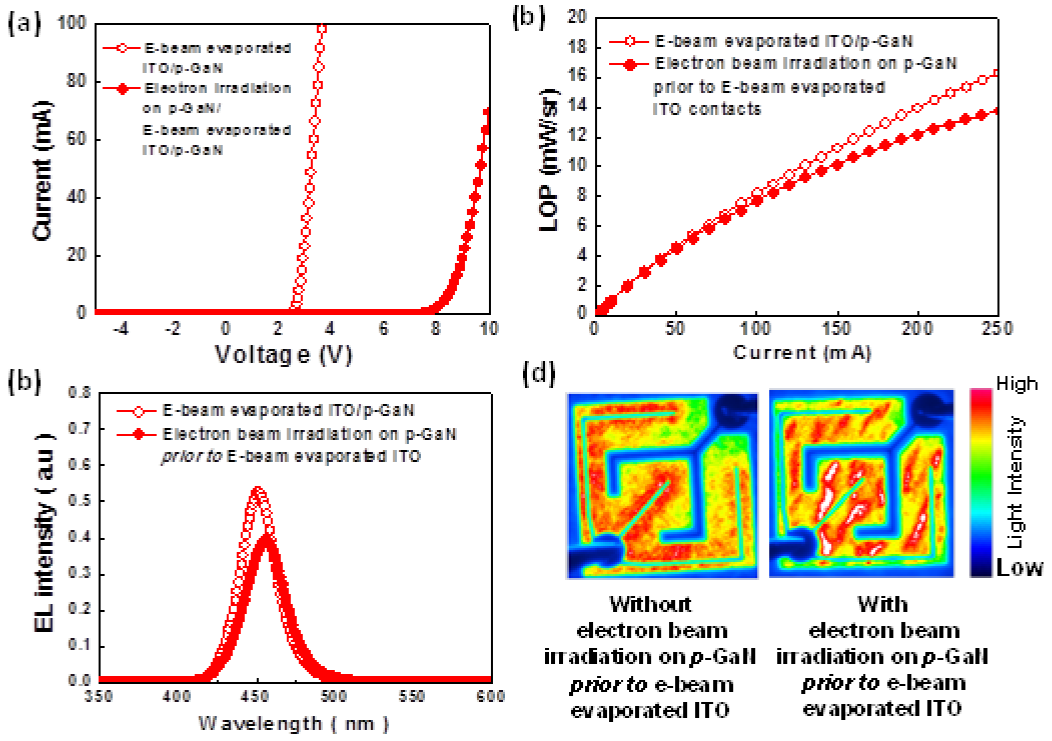

Figure S3. For the InGaN/GaN LEDs with and without the electron-beam irradiation on *p*-GaN by 2-inch electron-beam gun prior to the deposition of e-beam evaporated ITO electrode, a) *I*-*V* characteristics, b) LOP, c) EL intensity, and d) light intensity distribution images, were displayed.

S4. Fabrication of sputtered ITO Schottky diodes on *p*-GaN in the LED structure

We fabricated the Schottky diodes on *p*-GaN with the RF-sputtered ITO, and two-step-sputtered ITO with first contact layer (10 nm) using RF+DC (80 W+40 W) followed by the second RF-sputtered layer (50 nm) which was developed in this study. Four different diameters (60, 80, 100, 120 μm) of the sputtered ITO Schottky diodes were fabricated on p-GaN in the LED structures, where the Ni/Ag (2 nm/200 nm) was used as an ohmic contact on *p*-GaN, as shown in the following Figure. As shown in the I-V characteristic of the Ni/Ag contact on *p*-GaN showed a linear curve, indicating the formation of good ohmic contact, and the contact resistivity obtained from the TLM was $1x10^{-3}$ Ω-cm^2.

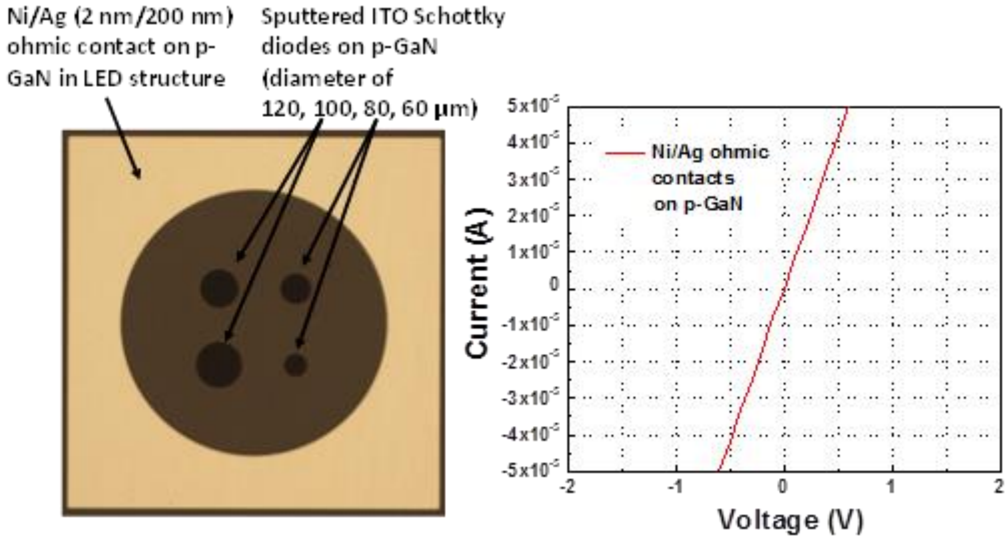

Figure S4. (Left) An optical microscope image of sputtered ITO Schottky diodes on p-GaN, and (right) I-V characteristic of the Ni/Ag ohmic contact on p-GaN, which shows the formation of good ohmic contact on p-GaN.

S5. Electron beam irradiation on *p*-GaN prior to e-beam-evaporated ITO *p*-electrodes

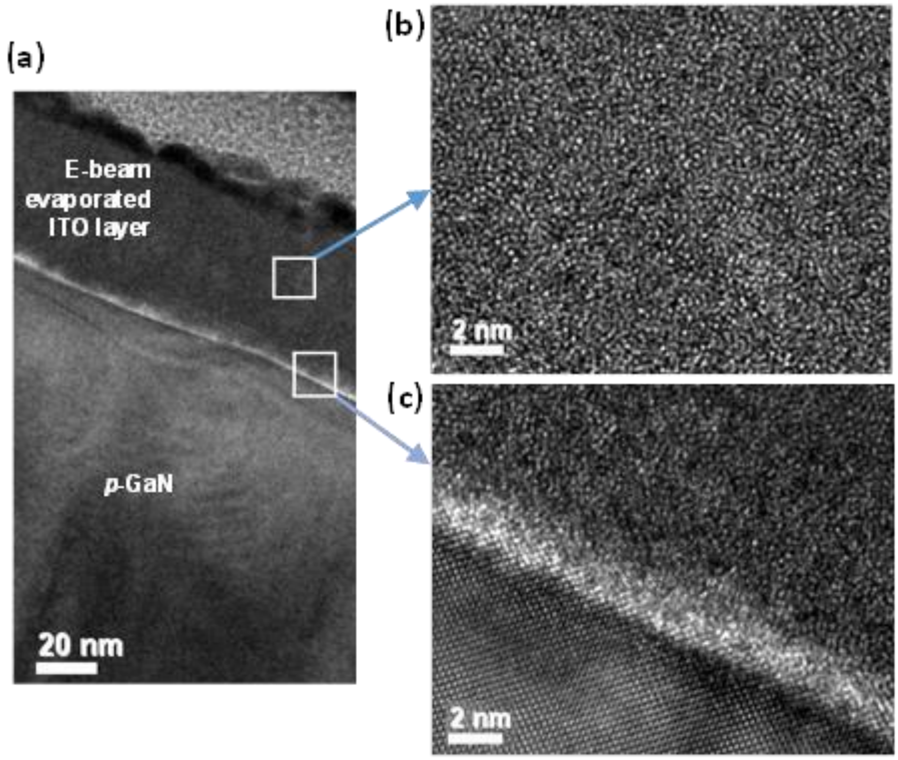

Figure S5. a) A low-magnification cross-sectional TEM image of the as-deposited e-beam evaporated ITO on *p*-GaN, b) high-magnification TEM image at the as-deposited e-beam evaporated ITO film, showing an amorphous crystal microstructure, and c) high-magnification TEM image at the interface between the as-deposited e-beam-evaporated ITO film and *p*-GaN, showing an amorphous crystal microstructure at the e-beam-evaporated ITO/*p*-GaN interface.
